# Effects of robot-assisted upper limb training combined with functional electrical stimulation in stroke patients: study protocol for a randomized controlled trial

**DOI:** 10.1186/s13063-024-08199-2

**Published:** 2024-06-04

**Authors:** Xu Yang, Wang Fengyi, Chen Yi, Qiu Lin, Lin Yang, Li Xize, Liu Shaxin, Yang Yonghong

**Affiliations:** 1 Rehabilitation Medicine Center, Sichuan University West China Hospital, Chengdu, 611135 Sichuan Province China; 2 Sichuan University West China School of Medicine, Chengdu, 611135 Sichuan Province China

**Keywords:** Robot-assisted training, Functional electrical stimulation, Stroke, Upper limb, Rehabilitation

## Abstract

**Introduction:**

About 17–80% stroke survivors experience the deficit of upper limb function, which strongly influences their independence and quality of life. Robot-assisted training and functional electrical stimulation are commonly used interventions in the rehabilitation of hemiplegia upper extremities, while the effect of their combination remains unclear. The aim of this trial is to explore the effect of robot-assisted upper limb training combined with functional electrical stimulation, in terms of neuromuscular rehabilitation, compared with robot-assisted upper limb training alone.

**Methods:**

Individuals (*n* = 60) with the first onset of stroke (more than 1 week and less than 1 year after stroke onset) will be considered in the recruitment of this single-blinded, three-arm randomized controlled trial. Participants will be allocated into three groups (robot-assisted training combined with functional electrical stimulation group, robot-assisted training group, and conventional rehabilitation therapies group) with a ratio of 1:1:1. All interventions will be executed for 45 min per session, one session per day, 5 sessions per week for 6 weeks. The neuromuscular function of the upper limb (Fugl-Meyer Assessment of upper extremity), ability of daily life (modified Barthel Index), pain (visual analogue scale), and quality of life (EQ-5D-5L) will be assessed at the baseline, at the end of this trial and after 3 months follow-up. Two-way repeated measures analysis of variance will be used to compare the outcomes if the data are normally distributed. Simple effects tests will be used for the further exploration of interaction effects by time and group. Scheirer-Ray-Hare test will be used if the data are not satisfied with normal distribution.

**Discussion:**

We expect this three-arm randomized controlled trial to explore the effectiveness of robot-assisted training combined with functional electrical stimulation in improving post-stroke upper limb function compared with robot-assisted training alone.

**Trial registration:**

Effect of upper limb robot on improving upper limb function after stroke, identifier: ChiCTR2300073279. Registered on 5 July 2023.

## Introduction

Stroke is one of the leading causes of death and disability around the world, which causes huge pressure on the global health system [1, 2]. Seventeen per cent to 80% of stroke survivors experienced the impairment of motor function of the upper limb, which significantly obstacles their independence and self-caring [3–5].

Conventional rehabilitation therapies (CRTs), mainly including task-oriented activities, activities of daily living (ADL) practice, instrumental activities of daily living (IADL) practice and constrain-induced movement therapy, is a widely-used and high-efficiency intervention in the recovery of upper limb function, which has been recommended in recent American Heart Association guidelines in class I [6]. However, its high human cost and time consumption always restrict patients to access to sufficient therapy [7].

An alternative approach is upper limb robot-assisted therapy (RT) as it can provide high-intensity and cost-effective training to reduce the pressure on human cost. One recent big-sample multicenter randomized controlled trial (RCT) and several systematic reviews reveal that RT is equivalent to CRTs in developing post-stroke upper limb neuromuscular function [8–10]. However, passive movement offered by robots does not provide sufficient activation and stimulation to hemiplegic muscles.

For further improving effectiveness, RT is combined with other therapeutic techniques like functional electrical stimulation (FES) [11, 12]. FES is a kind of neuromuscular electrical stimulation (NMES) generating muscle contraction during tasks or activities [13]. FES forces target muscle group contracting to improve the participation of patients and intensify the force and sensory feedback, which compensates for the disadvantage of RT. On the other hand, FES therapy requires patients to perform specific tasks during electrical stimulating, which is a huge challenge for stroke survivors. Supportive movement provided by robot promotes the completion of tasks. Considering this intrinsic complementary mechanism, Robot-assisted training combined with functional electrical stimulation (RTFE) seems a promising intervention in stroke function reconstruction. Nevertheless, scarce studies focus on the effectiveness of RTFE on post-stroke upper limb neuromuscular function. Existing RTFE studies mainly concentrate on developing FES controllers from an engineering perspective [14–16]. Most of them are small sample size and lack of comparison [14–16]. Katie’s research showed RTFE improve the motor function of the forearm; however, this trial, without the control group, only recruited 5 subjects [15]. Similarly, one trial investigated RTFE’s effect in stroke survivors but just on hand function with 5 subjects. Furthermore, these new-type RTFE system with advanced controllers seems not to be applied in clinical situations in a short time as they are mainly in the laboratory stage.

There is an urgency to conduct a clinical study to investigate the rehabilitative effectiveness of RTFE. The aim of this trial is to explore the effectiveness of RTFE on developing post-stroke upper limb neuromuscular function.

## Objective and hypothesis

The primary objective of this study is to investigate the effectiveness of RTFE on the upper extremity neuromuscular function in stroke patients. The null hypothesis is that there is no difference among participants who receive RTFE, RT and CRTs in terms of upper limb neuromuscular function development. The secondary objective is to investigate whether RTFE is superior to RT with respect to improving the ability of ADL, quality of life and reducing post-stroke pain.

## Methods

### Study design

**Fig. 1:**
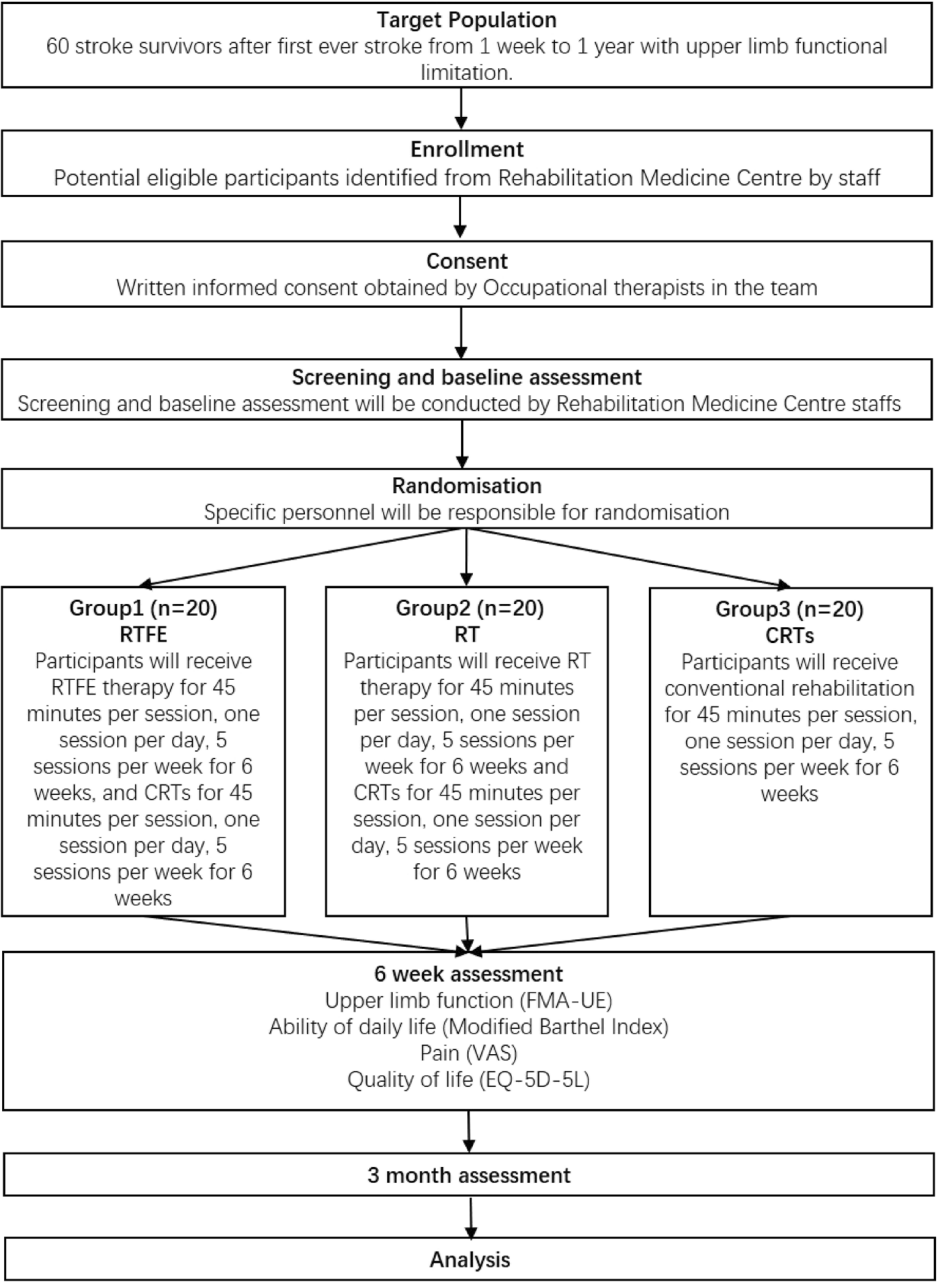
Flow chart of this trial

**Fig. 2:**
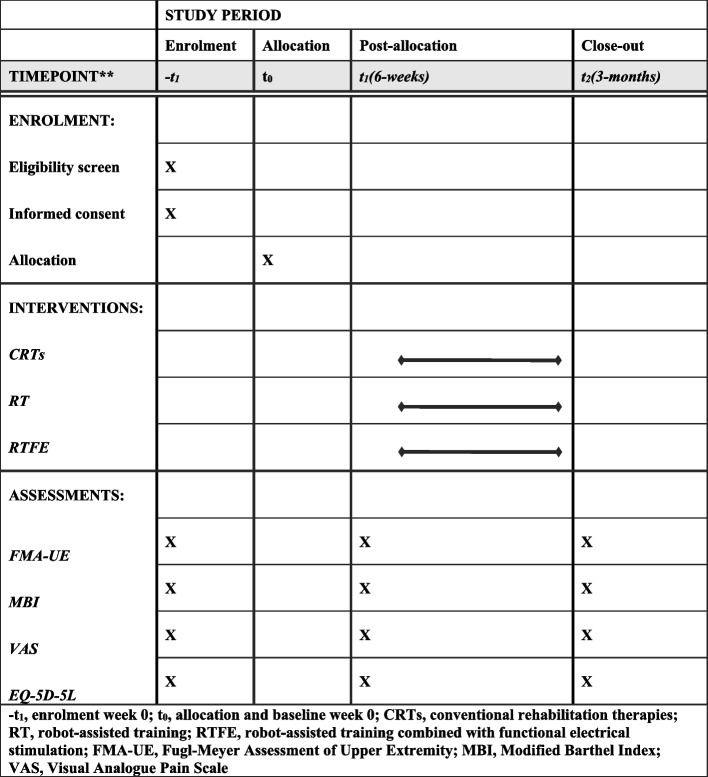
Example template of recommended content for the schedule of enrolment, interventions, and assessments*. − *t*_1_, enrolment week 0; *t*_0_, allocation and baseline week 0; CRTs, conventional rehabilitation therapies; RT, robot-assisted training; RTFE, robot-assisted training combined with functional electrical stimulation; FMA-UE, Fugl-Meyer Assessment of Upper Extremity; MBI, modified Barthel Index; VAS, visual analogue pain scale

This single-blind (assessor blinding), three-arm, superiority RCT will include 3 evaluation sessions (baseline, the end of interventions (6 weeks) and the end of follow-up (3 months)), and 6-week interventions for three groups (CRTs, RT and RTFE). The flow chart of this study is shown in Fig. 1. The SPIRIT figure is shown in Fig. 2.

This study will be conducted between 1st September 2023 and 1st March 2024 in the rehabilitation centre of West China Hospital of Sichuan University, Chengdu, Sichuan Province, China.

### Participants and sample size

Potentially eligible patients and people interested in this study will be appointed for eligible evaluation and further consent by well-experienced occupational therapists in the team. Eligible subjects will sign informed consent by the project leader XY if they are willing to participate in.

### Eligibility criteria

Participants presenting in this trial will be recruited based on the following criteria:

#### Inclusion criteria

- Aged 18 to 80
- Clinical diagnosis of stroke (cerebral infarction, primary intracerebral haemorrhage, subarachnoid haemorrhage, etc.)
- Between 1 week and 1 year since the first onset of stroke
- Upper limb, functional limitation: the score of Fugl-Meyer Assessment of Upper Extremity (FMA-UE) ≥ 11 and ≤ 33
- All of the following muscle strengths assessed by manual muscle test (shoulder flexion/extension, shoulder abduction/adduction, elbow extension/flexion, wrist flexion/extension) ≤ 3
- Sufficient to offer consent to take part in this study and able to follow the requirements of the protocol

#### Exclusion criteria

- The history of more than one stroke
- Other current significant upper extremity impairment, e.g., frozen shoulder, fracture
- Previous any kinds of robot-assisted rehabilitation experience
- Enrolment in another clinical trial
- Accepted upper limb botox injection, isokinetic training, and electrotherapy within 3 months

#### Discontinuing criteria

Withdrawing will be in consideration if:

- Participants decide to withdraw
- Participants develop serious diseases such as serious heart attacks and stroke
- Therapists, stroke physicians and investigators may also withdraw participants if they think this trial is no longer in patients’ interest; for example, continuing this trial might induce more adverse events

### Sample size

The sample size is 60 subjects (20 subjects in each group). As we did not retrieve an appropriate study, according to Cohen’s theory [17], the effect size of 0.25 was applied. G*Power was used to estimate the sample size (repeated measures analysis of variance (ANOVA), within-between interaction) with effect size = 0.25, *α* = 0.05, 1 − β = 0.95, and the number of groups = 3. The total sample size based on the result was 54 (18 subjects per group). Considering 10% attrition, we inflated the total sample size to 60.

### Randomization and blinding

Participants will be randomized to the RTFE group, RT group and CRTs group with 1:1:1 allocation as per a computer-generated randomization schedule stratified by time after stroke (< 3 months versus ≥ 3 months) using permuted blocks of random size. The size of the block will not be disclosed to ensure concealment. The randomization schedule will be generated by a physical therapist (LXZ).

Pre-prepared opaque, sequentially numbered, sealed envelopes will be used to assign all participants into three groups. The printed information of allocation will be separately stored in envelopes. LXZ will make these envelopes and sign the back of them after sealing. All envelopes will not be open until corresponding participants are enrolled.

Recruitment and assignment will be conducted by LY who will not participate in the outcome assessment and statistics process. Participants in this trial will be required not to reveal their allocations and therapy contents to the assessors and other participants.

In this trial, all evaluators, a part of intervention implementers, and people in charge of statistical analysis will be blinded. Unblinding is allowable only when a serious adverse event occurs.

## Interventions

There will be three groups (RTFE group, RT group, and CRTs group) in this study. Participants in the CRT group will receive only conventional rehabilitation therapies. Participants in the RT group will receive robotic training with CRTs and a sham FES. Participants in the RTFE group will receive robotic training with FES and CRTs. The duration of CRTs in each group will be the same, and RT in both the RT group and the RTFE group will be set based on the same method in the Appendix.

During this experiment, all participants will be required to maintain the original lifestyle and try not to seek for other upper limb-related medical care such as extra kinesitherapy, which is for reducing the impact of adjusting lifestyle or different amounts of training.

### CRT group (conventional rehabilitation therapies group)

Eligible participants in the CRTs group will be treated with daily rehabilitative training and clinical pharmacotherapy. All training will be set based on the participants' functional situation and aim to develop upper limb function. The rehabilitative training mainly includes functional training, manual training, coordination training, mental practice and task-oriented training. Additionally, all therapists will be required to track and record all specific rehabilitation programmes with detailed information such as duration and frequency. Standard CRTs will be conducted for 45 min per session, one session per day, 5 days per week, for 6 weeks.

### RT group (robot-assisted training group)

Participants in the RT group will receive both RT and CRTs. The Upper Limb Training and Evaluation System (A6) which is produced by Guangzhou Yikang Medical Equipment Industrial Co., LTD, used in the RT programme consists of an exoskeleton with 3 modules (shoulder module, elbow module, and wrist module), a laptop and a screen. It will support hemiplegic individuals to perform passive, partially active, and totally active upper limb movements of shoulder flexion/extension, shoulder abduction/adduction, elbow flexion/extension, and wrist flexion/extension. This machine could also provide simulating ADL tasks such as drinking, feeding, and combing hair with different levels of assistance. The screen provides visual and audio feedback by showing a virtual character doing the same action with users, and sounding if a particular movement is successfully performed. Specifically trained occupational therapists will choose an individualized RT training plan consisting of several motions for each participant based on his/her functional status. The process of making the RT programme has been shown in the Appendix. Participants in the RT group will receive sham FES. For the sham stimulation, the stimulator will be turned on but the intensity button will be set to 0; thus, there will be no output. Participants in the RT group will be notified that the stimulation will have been set below the sensory threshold. Participants will receive RT training for 45 min per session, one session per day, 5 sessions per week, for 6 weeks. Participants in the RT group will also receive the CRTs as same as in the CRT group.

### RTFE group (robot-assisted training combined with functional electrical stimulation group)

Participants in the RTFE group will receive CRTs and RTFE. Both CRTs and RT will be as same as those in the RT group. Extra FES will be set on people in the RTFE group. FES (Dualpex-071 Quark Medical, Brazil) will be applied during the RT for 45 min by the surface electrodes. The position of surface electrodes will be set on the surface of muscle involved in the RT movement, which primarily includes the deltoid, triceps brachii, biceps brachii, wrist flexor and wrist extensor muscle of the paretic arm. Stimulation parameters will be set with (1) frequency = 40 Hz, (2) pulse width = 300 μs, (3) on time (contraction = 8 s), (4) off time = 2 × on time, and (5) current intensity = the maximum tolerance of participant. These parameters have been set considering a previous study showing a positive change in reaching motor performance in stroke survivors [18]. The FES will be given synchronously with robot movement. The intensity of FES will be set to the maximum which can be tolerated by participants. FES will be conducted simultaneously when the RTFE participants accept the RT.

## Outcomes

### Primary outcome

#### FMA-UE (Fugl-Meyer Assessment of Upper Extremity)

The primary outcome of this study is the upper limb neuromuscular function measured by FMA-UE at the end of week 6. FMA-UE is an assessment of the upper extremity in terms of neuromuscular function with high intra- and inter-reliability and concurrent validity [19, 20]. This scale consists of 33 items, which mainly focus on the reflex activity, volitional movement and functional movement of the upper extremity, wrist and hand, with the score of each item ranging from 0 to 2 (0 = no performance at all; 1 = partial performance; 2 = full performance). The maximum score for this assessment is 66 and the minimal clinically important differences are 12.4 (upper extremity), 5.6 (upper arm), and 4.9 (wrist/hand) [21]. A higher FMA-UE score means better performance in neuromuscular function.

### Secondary outcomes

The secondary outcomes include ADL measured by the modified Barthel index (MBI), upper limb pain intensity assessed by the visual analogue scale (VAS), and quality of life assessed by EuroQol 5-Dimension 5-Level (EQ-5D-5L).

#### MBI (modified Barthel Index)

ADL will be assessed by the MBI, which has shown good validity, reliability and sensitivity in Chinese stroke patients’ ADL assessment [22]. MBI is a functional scale consisting of 10 items including feeding, bowel control, bladder control, personal hygiene, transfer, dressing, toilet ambulation, bathing and stairing, and each item in MBI is categorized into 5 levels. The final score of MBI ranges from 0 to 100, of which a higher score indicates a better capacity of independence.

#### VAS (visual analogue scale)

The upper limb pain intensity in our study will be captured by a 0–10 visual analogue pain scale. According to their severity of pain, participants will be required to mark on a 100-mm line. The far left of the line represents no pain and the far right means the worst possible pain. The score of VAS will be represented as the number corresponding to the marked position. VAS shows good intra- and inter-reliability and validity across many experiments and different populations [23].

#### EQ-5D-5L (EuroQol 5-Dimension 5-Level)

Quality of life will be assessed by EQ-5D-5L. EQ-5D-5L is a measurement of quality of life based on 5 items of mobility, self-care, usual activities, pain/discomfort, and anxiety/depression. The score of EQ-5D-5L is between 0 (dead) to 1 (the best quality of life). Its reliability and validity in stroke patients have been discussed in previous articles [24–26].

Demographic details will be collected. These will include (1) age, (2) sex, (3) diagnosis: ischemic stroke or hemorrhagic stroke, (4) time of the first onset of stroke, (5) the side of the affected arm, and (6) Brunnstrom stage.

## Statistical methods

Normally distributed variables will be presented by means with standard deviations, and nonnormally distributed data will be presented by medians with interquartile ranges. Categorical variables will be presented by proportion and frequency.

Investigators in charge of the result analysis will be blinded to the group allocation. All data will be analysed by Statistical Package for the Social Science (SPSS) version 26.0 software. The score of the primary outcome and secondary outcomes will be measured at different time points for the same subject. Two-factor repeated-measures analysis of variance (ANOVA) will be used for the statistical analysis for all outcomes with the sphericity test before analysis. If the data is dissatisfied with the sphericity test, the Green-house-Geisser method will be used to correct it. Simple effects tests will be used for the further exploration of interaction effects by time and group. Scheirer-Ray-Hare test will be used if the data are not satisfied with normal distribution. The statistical difference will be set at a *P* value less than 0.05.

The subgroup analysis will be conducted for further exploration. The comparison will be conducted between people with the first stroke onset for less than three months and residual people in terms of the score of FMA-UE. We will test whether RTFE shows the same effectiveness in people with different durations after first stroke onset by two-factor repeated measures ANOVA with the same statistical difference.

All analyses in this trial will follow the intention-to-treat principle. Based on this principle, all participants no matter what kind of intervention they received will be regarded as randomized. Multiple imputations based on 5 replications and the Markov-chain Monte Carlo method in the SAS MI procedure will be used to handle the missing data.

## Data collection and management

### Data collection and storage

Outcomes will be measured by two certified, well-experienced occupational therapists who will neither be informed of the allocation nor participant in any interventions. The participants will receive assessments of the FMA-UE, MBI, VAS and EQ-5D-5L at baseline, after completion of their interventions (6 weeks), and at the end of follow-up (3 months). Case report forms (CRFs) will be used to record participants’ clinical information. The corresponding questionnaires will be used to record the results of the assessment. These paper-based data will be transferred into Microsoft Excel 2020 and EpiData 3.1 software for good management. Two independent researchers will take charge of double data entry and verification to guarantee the accuracy and truthfulness of the data. The original data will be checked again for further verification if there is inconsistent data inputting or missing. All data will not be allowed to be modified after input and check.

Personal data will be strictly confidential. The original paper CRFs of participants will be locked in a file cabinet at the research site. Coded ID numbers will be used in all reports, data collection, processing and administrative forms for keeping participants’ confidentiality. All paper records with names or personal identifiers will be stored separately from documents with a coded number. The data will be stored in a computer with passwords set by the leader of this project at the research site, and only staff involved in this study will be allowed to access it. The personal information of participants will not be released without the written permission of the participant.

### Retention strategies

The following strategies will be conducted to promote retention and follow-up completion. Before the beginning of this study, all participants will be informed of the process of this trial in detail by two research assistants to ensure that they can fully understand the process of this trial. Participants can question during the whole trial and the leader of this trial will provide answers. An online brief interview on Zoom between research assistants and participants will be held by these research assistants once a week to improve the adherence of participants. Periodic reports about the progress of this study will be made to maintain the interest of patients. The reports will be transferred by WeChat or email. Before the final data collection, a reminder will be transferred by WeChat or email to remind participants of the upcoming assessment.

## Monitoring

WFY (the project leader), YYH (an expert in clinical statistics) and XY (be responsible to the study conduction) will constitute the group of the coordinating centre. The trial steering committee will consist of three staff from the Sichuan University for data supervision, who will not be involved in this trial.

The formal interim analysis will not be performed for the following reasons: (1) the expected risk of this trial is low; (2) the goal of this trial is explicit, which is able to guarantee the smooth implementation of this trial.

In this trial, an adverse event will be defined as any untoward medical occurrence in a subject. This trial will not use investigational medicinal products. All participants will be monitored during the intervention and all adverse events will be recorded and reported. If serious adverse events occur, all interventions will be stopped immediately and participants will receive appropriate treatments. Serious adverse events will be reported to the Ethics Committee of West China Hospital of Sichuan University.

Frequency and plans for auditing trial conduct and the progress of this study will be reported by the coordinating centre. The procedure of this trial will be regularly supervised by the trial steering committee and ethics committee every 4 weeks. Recommendations of the protocol will be given if necessary. On-site monitoring will be used to review the trial process.

## Discussion

We suppose that this trial will reveal the effectiveness of RTFE on upper limb dysfunction after stroke. One experiment conducted by Meadmore investigated the FES mediated by iterative learning control plus robotics in reducing motor impairment in 5 chronic stroke patients [15]. A significant improvement was observed after 18 RT sessions in subjects. Our RCT will provide evidence of comparison among three groups and further explore the reliability of this conclusion with a bigger sample size. Although FES in our study is based on the open-loop design, considering this is the most widely used FES and most FES with new-type controllers are still immature, this might be the easiest protocol to implement, which makes the result of our trial with high practical value. Straudi’s recent study investigated robot-assisted hand training with FES in improving the hand function of stroke patients [11]. The result shows an equivalent improvement between the RTFE group and the CRT group. Our trial focuses on the proximal joints of the upper limb, and the result of this trial will reveal the influence of RTFE on shoulder, elbow, and wrist neuromuscular function after stroke.

There are several strengths and weaknesses of this trial. Firstly, compared with previous self-controlled study, this study is a well-structured 3-arm RCT. Our study compares the effect between RTFE with RT alone to explore the overlapping effect of this combination therapy. Then, this study adopts various methods to reduce the bias and enhance the reliability as possible. Considering the difference of neuroplasticity potential in stroke survivors at different stages, stratification by baseline of time after the first-ever stroke is used to reduce the heterogeneity among the three groups. The use of a randomized block design reduces the bias and promotes balance in the allocation. The systematic imputation strategies further guarantee data and conclusion’s reliability. Additionally, the use of intention-to-treat analysis reduces the selection bias. Although not all interveners and participants are blinded, the blinding still covers assessors, RT therapists, CRT therapists and participants in the RT and RTFE groups for reducing potential bias.

The main weakness of this trial is that the participant recruitment just covers stroke survivors with moderate and severe motor deficits (FMA-UE ≤ 33), so the conclusion of this trial is not able to be applied to stroke survivors with motor function. Future studies could focus on the application of RTFE in the broader population.

## Conclusion

The study aims to investigate the feasibility and effectiveness of RTFE in stroke upper limb neuromuscular function. The result may contribute to optimize existing upper limb RT.

### Trial status

The protocol is in version 2.0. No patient has been recruited in this trial at the time of submission of this manuscript. This trial has not been started and the approximate date of completion is expected on 1st March 2024.

## Appendix

**Table:**

Item	Robot-assisted training
Materials	The upper limb Training and Evaluation System (A6) is used ( href="http://www.yikangshiye.com/upper/a6.html" data-mce-href="http://www.yikangshiye.com/upper/a6.html">http://www.yikangshiye.com/upper/a6.html) in RT in this trail. The robotic system consists three modules (shoulder module, elbow module, and wrist module) to improve participants’ upper extremity function. The shoulder module: The movements of participants’ shoulder include flexion, extension, horizontal abduction, horizontal adduction. The elbow module: The module supports affected elbow joint flex and extend. The wrist module: This module integrated onto the elbow module, which encouraging the affected wrist flex and extend.
Procedures	Staff delivering the robot-assisted training receive specific training. The robot-assisted training consists of two patterns (passive pattern and partially active pattern) in each module. Passive pattern: used in joints with muscle strength = 0 and 1 (assessed by MMT by a senior therapist). In passive pattern, the module rhythmically moves the participants’ upper limb to reach targets in sequence. Partially active pattern: used in joints with muscle strength range from 2 to 3. In this pattern, the robot provides partial supports to move participants’ upper limb to reach sequentially presented targets, and the participants are required to finish the task in their best effort. The supportive level is set by the senor therapist received specific training. Each programme includes 3 separative parts: shoulder movement, elbow movement and wrist movement, and each part lasts 15 min. The trained senior therapist choose suited pattern of each movement to make the final programme of each participants. Considering the individuals’ function changing during the intervention, appropriate upgrading and downgrading would be conducted and recorded. For example; individuals perform better in shoulder passive adduction/abduction with muscle strength growth would be upgraded to shoulder adduction/abduction in partial active pattern in remaining time.
Who provider?	A senior therapist assesses participants at the initial session to guarantee the correct positioning. Therapy assistants then conduct the training under the supervision of senior therapist. The senior therapist also reviews all participants every week and provide feedback and suggestions. Both senior therapist and therapy assistants receive specific training in this RT assessment and conduction.
When and how much?	The RT training programme is set for 45 min per session per day, 5 sessions per week for 6 weeks.

*RT *Robot-assisted training

## Abbreviations

CRTs: Conventional rehabilitation therapies
ADL: Ability of daily life
IADL: Instrumental activities of daily living
RT: Robot-assisted training
RCT: Randomized controlled trial
FES: Functional electrical stimulation
NMES: Neuromuscular electrical stimulation
RTFE: Robot-assisted training combined with functional electrical stimulation
FMA-UE: Fugl-Meyer Assessment of Upper Extremity
MBI: Modified Barthel Index
VAS: Visual analogue pain scale
ANOVA: Analysis of variance
CRFs: Case report forms
